# Decrease in late presentation for HIV care in Kinshasa, DRC, 2006–2020

**DOI:** 10.1186/s12981-021-00366-8

**Published:** 2021-07-16

**Authors:** Nadine Mayasi Ngongo, Hippolyte Situakibanza Nani-Tuma, Marcel Mbula Mambimbi, Murielle Longokolo Mashi, Ben Bepouka Izizag, Faustin Kitetele Ndolumingu, Nathalie Maes, Michel Moutschen, Gilles Darcis

**Affiliations:** 1 Department of Internal Medicine, Infectious and Tropical Diseases, University Clinics of Kinshasa, Kinshasa, Democratic Republic of the Congo; 2 Biostatistics and Medico-economic Information Department, University Hospital of Liege, Liège, Belgium; 3 Department of Internal Medicine and Infectious Diseases, Liège University Hospital, Liège, Belgium; 4 AIDS reference laboratory, University of Liège, Liège, Belgium

**Keywords:** Late presentation, HIV care, Kinshasa

## Abstract

**Introduction:**

Late presentation for HIV care is a well-described issue for the success of ART outcomes and the cause of higher morbidity, mortality and further transmission. Monitoring the level of late presentation and understanding the factors associated with it would help to tailor screening and information strategies for better efficiency.

We performed a retrospective cohort study in Kinshasa, the capital of the DRC. The studied population included HIV-positive adults newly enrolled in HIV care between January 2006 and June 2020 at 25 HIV urban care facilities. Patient information collected at presentation for HIV care included age, sex, WHO clinical stage and screening context. We used 2 definitions of late presentation: the WHO definition of advanced HIV disease (WHO stage 3/4 or CD4 cell count < 200 cells/mm^3^) and a more inclusive definition (WHO stage 3/4 or CD4 cell count < 350 cells/mm^3^).

**Results:**

A total of 10,137 HIV-infected individuals were included in the analysis. The median age was 40 years; 68% were female. A total of 45.9% or 47.5% of the patients were late presenters, depending on the definition used. The percentage of patients with late presentation (defined as WHO stage 3/4 or CD4 cell count < 350 cells/mm^3^) decreased during recent years, from 70.7% in 2013 to 46.5% in 2017 and 23.4% in 2020. Age was associated with a significantly higher risk of LP (p < 0.0001). We did not observe any impact of sex.

**Conclusions:**

The frequency of late presentation for care is decreasing in Kinshasa, DRC. Efforts have to be continued. In particular, the issue of late diagnosis in older individuals should be addressed.

## Introduction

Antiretroviral therapy (ART) represents a remarkable success in recent medicine. ART radically decreases the morbidity and mortality of human immunodeficiency virus (HIV)-infected individuals and drastically reduces the risk of HIV transmission [1, 2].

The development of effective and less toxic medications led to guideline modifications culminating in 2015 with the adoption of the “treat all” strategy. For the first time, the WHO guidelines recommended that ART should be initiated in everyone living with HIV with any CD4 cell count [3]. The 2017 consolidated guidelines stated that ART initiation should be offered on the same day to people who are ready to start, with the worthy objective of improving the scaling up of ART [4].

The implementation of the WHO and national guidelines led to a significant decrease in time from diagnosis to treatment initiation, as shown in multiple studies in Sub-Saharan Africa [5–7]. Although this observation shows the success of the treat all strategy implementation, it should not mask the fact that many other hurdles remain to reach better ART coverage.

By the end of 2019, it was estimated that 67% of people living with HIV worldwide were on ART [8]. In Africa, this number reached 70% [8]. The numbers are increasing over time. Nevertheless, many infected individuals still ignore their status. Among them, many will likely present for care later in the course of the infection.

Indeed, late presentation (LP) for care is a well-described, persistent issue for the success of ART outcomes. Late presenters suffer from a higher morbidity and mortality and from a poorer immune recovery [9, 10]. Late presenters can also transmit HIV during a prolonged period. Moreover, LP could be associated with a higher chance of attrition from care during follow-up [11]. From a cure perspective, it is well established that early ART initiation is associated with an HIV latent reservoir of smaller size and reduced diversity, a phenotype that would favor several cure strategies [12, 13].

Despite its recognized negative impact on the HIV epidemic, late presentation for care remains a too-common concern in many regions worldwide [14–19]. Various strategies can be used to mitigate the LP issue, including information campaigns, HIV screening promotion, and combating stigma and discrimination.

Understanding the factors associated with late presentation in a local context would help to tailor screening/information strategies for better efficiency.

Here, we studied the frequency of LP over time in Kinshasa, Democratic Republic of Congo (DRC). We also performed an analysis of the factors associated with LP with the goal of refining local screening policies.

## Material and methods

### Study design and analysis

This was a retrospective cohort study in Kinshasa, the capital of the DRC. The studied population included HIV-positive adults (≥ 16 years) newly enrolled in HIV care between January 2006 and June 2020 at 25 HIV urban care facilities. The participating facilities are part of national ART programs, and the provision of services at each facility was conducted according to the guidelines of the Congolese Ministry of Health and the WHO. Patient information routinely collected at presentation for HIV care included age, sex, WHO clinical stage and screening context. Regarding the screening context, HIV diagnosis was made either at Kalembe Lembe Pediatric Hospital (KLL, services to HIV^+^ children and their first-line family members) or at various centers related to the International Centre for Aids Care and Treatment Programs (ICAP). As children were not included in the analysis, only family members diagnosed at KLL were included.

We used two definitions of late presentation:

- The WHO definition of advanced HIV disease: a CD4 cell count of < 200 cells/mm^3^ or a WHO clinical stage 3 or 4 event at presentation for care [4],
- A more inclusive and widely used definition in the literature [19, 20]: < 350 cells/mm^3^ or a WHO clinical stage 3 or 4 event at presentation for care.

We defined CD4 count testing at presentation as having a CD4 cell count performed up to 3 months following HIV diagnosis.

The data for this study were collected either directly from the standard patient medical records or from the electronic databases, depending on the facility. Data quality assessments were performed every 6 months to assess the completeness and accuracy of data entry. The Ethical Committee of the School of Public Health, University of Kinshasa approved the use of data for this study.

### Statistical analysis

Quantitative variables are summarized as the mean and standard deviation (SD) or median and interquartile range (IQR: p25–p75). Categorical variables are presented as frequency tables (numbers and percentages). The impact of time (year of HIV diagnosis) and other factors on baseline CD4 count testing were analyzed using logistic regression models. No missing data was replaced. The results were considered significant at the uncertainty level of 5% (p < 0.05). The statistical analyses were performed using SAS software (version 9.4), and the graphics were made using R software (version 3.6.1).

## Results

A total of 10,137 HIV-infected individuals were included in the analysis (Table 1). The median age was 40 years, 68% were female, and 91% were married. The median body mass index (BMI) was 21.9. The majority of HIV diagnoses were made at ICAP centers (89%). The number of days between diagnosis and initial CD4 cell count was short (mean ± SD: 1.6 ± 14.8).

**Table 1:** Characteristics of HIV^+^ individuals at time of HIV diagnosis (N = 10137)

	N (%)	Mean ± SD	Median (IQR)	Extremes
Age (years)	10137	39.9 ± 11.0	40 (32; 47)	16–88
Sex (female)	6925/10137 (68.3)			
Height (cm)	1197	163 ± 8	163 (158; 168)	126–195
Weight (kg)	8762	60.0 ± 12.2	59 (52; 66)	30–120
BMI (kg/m^2^)	1197	22.6 ± 4.4	21.9 (19.6; 25.1)	11.2–42.7
Married	499/547 (91.2)			
Year of diagnosis				
2006	95/10137 (1.0)			
2007	103/10137 (1.0)			
2008	101/10137 (1.0)			
2009	192/10137 (1.9)			
2010	246/10137 (2.4)			
2011	311/10137 (3.1)			
2012	419/10137 (4.1)			
2013	576/10137 (5.7)			
2014	717/10137 (7.1)			
2015	705/10137 (7.0)			
2016	907/10137 (9.0)			
2017	1179/10137 (11.6)			
2018	1951/10137 (19.3)			
2019	1955/10137 (19.3)			
2020	680/10137 (6.7)			
Context of screening				
Kalembe Lembe Pediatric Hospital (KLL)	1099/10137 (10.8)			
International Centre for Aids Care and Treatment Programs (ICAP)	9038/10137 (89.2)			

As indicated in Table 2, 45.9% or 47.5% of the patients were late presenters, depending on the definition used. The difference between definitions is slight because the numbers were mainly influenced by WHO stages. Indeed, CD4 cell counts at baseline were rarely performed in the DRC and have been totally abandoned in recent years [21].

**Table 2:** Numbers and percentages of LP over time

	N	CD4 < 200/mm^3^ or WHO 3 or 4	CD4 < 350/mm^3^ or WHO 3 or 4
Year of diagnosis			
2006	94	67 (71.3)	71 (75.5)
2007	93	66 (71.0)	69 (74.2)
2008	95	58 (61.0)	64 (67.4)
2009	180	88 (48.9)	97 (53.9)
2010	238	125 (52.5)	143 (60.1)
2011	301	181 (60.1)	196 (65.1)
2012	403	244 (60.6)	262 (65.0)
2013	550	373 (67.8)	389 (70.7)
2014	688	449 (65.3)	467 (67.9)
2015	684	434 (63.4)	462 (67.5)
2016	855	496 (58.0)	519 (60.7)
2017	1141	529 (46.4)	531 (46.5)
2018	1927	760 (39.4)	760 (39.4)
2019	1944	498 (25.6)	498 (25.6)
2020	667	156 (23.4)	156 (23.4)
**All**	**9860**	**4524 (45.9)**	**4684 (47.5)**

**Figure 1:**
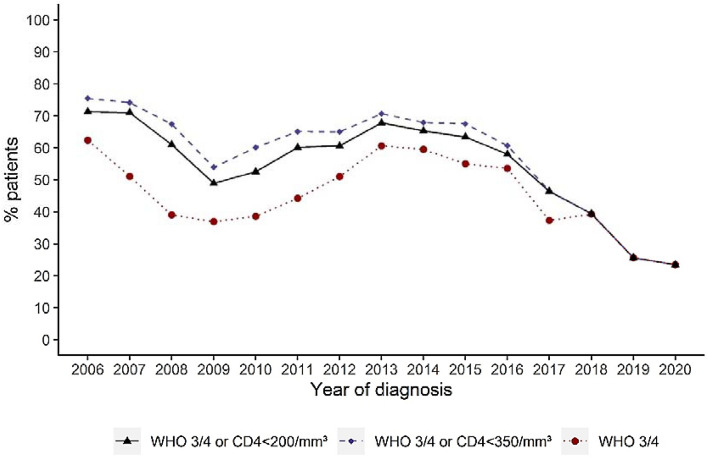
Percentage of LP over time.

It should be noted that the percentage of LP has decreased over time, particularly in recent years. We observed a drop from 70.7% in 2013 to 46.5% in 2017, 25.6% in 2019 and 23.4% in 2020; late presentation was defined as < 350 cells/mm^3^ or a WHO clinical stage 3 or 4 event at presentation for care (Table 2, Fig. 1).

### Factors independently associated with late presentation

The impacts of time (year of HIV diagnosis) and other factors on the probability of late presentation were analyzed using multiple logistic regression models. Table 3 presents the adjusted odds ratios of being a late presenter depending on the LP definition. Age was associated with a significantly higher risk of being a late presenter (p < 0.0001). The percentage of LP decreased over time (p < 0.0001). It should be noted that the probability of being a late presenter increased when a CD4 cell count at baseline was performed. This could be an important confounding factor in our analysis, as CD4 cell count measurement at baseline has been abandoned in recent years [21]. However, the percentage of LP based on a pure clinical definition also significantly decreased over time (Fig. 1, Table 3). Table 3 indeed shows that the risk of being a late presenter decreased over time when considering a pure clinical definition (OR: 0.79 (0.78-0.81); p < 0.0001). The decrease in HIV diagnosis at a late clinical stage is also illustrated in Fig. 1.

**Table 3:** Factors associated with late presentation—Multiple logistic regression model (N = 9860)

	Variables	Coefficient ± SE	OR (95% CI)	p-value
Probability of late	Intercept	0.058 ± 0.13	–	–
presentation (CD4 < 200/mm^3^	Age (by 10 years older)	0.22 ± 0.020	1.2 (1.2–1.3)	< 0.0001
or WHO 3 or 4)	Sex (ref = male)	− 0.0085 ± 0.024	0.98 (0.90–1.1)	0.72
	Year of HIV diagnosis (by year from 2006)	− 0.20 ± 0.010	0.82 (0.80–0.84)	< 0.0001
	Context of screening (ref = pediatric center KLL)	0.86 ± 0.045	5.6 (4.7–6.7)	< 0.0001
	Presence of CD4 measurement (ref = No)	0.86 ± 0.066	2.4 (2.1–2.7)	< 0.0001
Probability of late	Intercept	0.063 ± 0.13	–	–
presentation (CD4 < 350/mm^3^	Age (by 10 years older)	0.22 ± 0.021	1.3 (1.2–1.3)	< 0.0001
or WHO 3 or 4)	Sex (ref = male)	− 0.0087 ± 0.024	0.98 (0.89–1.1)	0.72
	Year of HIV diagnosis (by year from 2006)	− 0.20 ± 0.010	0.82 (0.80–0.84)	< 0.0001
	Context of screening (ref = pediatric center KLL)	0.82 ± 0.046	5.2 (4.3–6.2)	< 0.0001
	Presence of CD4 measurement (ref = No)	1.1 ± 0.069	3.1 (2.7–3.5)	< 0.0001
Probability of late	Intercept	0.35 ± 0.11	–	–
presentation (WHO 3 or 4)	Age (by 10 years older)	0.22 ± 0.020	1.2 (1.2–1.3)	< 0.0001
	Sex (ref = male)	− 0.0057 ± 0.024	0.99 (0.90–1.1)	0.81
	Year of HIV diagnosis (by year from 2006)	− 0.23 ± 0.0091	0.79 (0.78–0.81)	< 0.0001
	Context of screening (ref = pediatric center KLL)	1.1 ± 0.049	8.3 (6.9–10.1)	< 0.0001

In particular, the level of HIV^+^ individuals at WHO stage 1 radically increased over time, while the percentages of individuals diagnosed at later clinical stages (WHO 3 or 4) significantly decreased (Table 4). We did not observe any impact of sex on LP. In contrast, we showed an important impact of the type of center where HIV screening was offered, KLL vs ICAP. This was not unexpected, as screening at KLL is mostly performed on a systematic basis and largely targets asymptomatic individuals.

**Table 4:** WHO stage at HIV diagnosis

Year of diagnosis	N	WHO Stage				
		Non-missing n (%)	1	2	3	4
2006	95	93 (97.9)	15 (16.1)	20 (21.5)	49 (52.7)	9 (9.7)
2007	103	90 (87.4)	16 (17.8)	28 (31.1)	39 (43.3)	7 (7.8)
2008	101	95 (94.1)	38 (40.0)	20 (21.0)	30 (31.6)	7 (7.4)
2009	192	179 (93.2)	85 (47.5)	28 (15.6)	62 (34.6)	4 (2.2)
2010	246	223 (90.6)	97 (43.5)	40 (17.9)	73 (32.7)	13 (5.8)
2011	311	292 (93.9)	109 (37.3)	54 (18.5)	116 (39.7)	13 (4.4)
2012	419	380 (90.7)	128 (33.7)	58 (15.3)	168 (44.2)	26 (6.8)
2013	576	535 (92.9)	101 (18.9)	110 (20.6)	288 (53.8)	36 (6.7)
2014	717	669 (93.3)	164 (24.5)	107 (16.0)	365 (54.6)	33 (4.9)
2015	705	675 (95.7)	154 (22.8)	150 (22.2)	333 (49.3)	38 (5.6)
2016	907	849 (93.6)	224 (26.4)	170 (20.0)	408 (48.1)	47 (5.5)
2017	1179	1141 (96.8)	337 (29.5)	278 (24.4)	450 (39.4)	76 (6.7)
2018	1951	1927 (98.8)	668 (34.7)	501 (26.0)	670 (34.8)	88 (4.6)
2019	1955	1944 (99.4)	791 (40.7)	655 (33.7)	450 (23.1)	48 (2.5)
2020	680	667 (98.1)	317 (47.5)	194 (29.1)	141 (21.1)	15 (2.2)
All	10137	9759 (96.3)	3244 (33.2)	2413 (24.7)	3642 (37.3)	460 (4.7)

## Discussion

The development of effective and minimally toxic ART led to the adoption of the treat all strategy. The benefits of early ART have been strongly demonstrated at the individual and community levels. To reach the worthy objective of treating all HIV-infected individuals early, ART should be initiated soon after the diagnosis, as recommended. However, offering ART early during the course of the infection also requires early diagnosis.

Late presentation is indeed a persistent global concern. Factors associated with late presentation vary from one country to another and are affected by numerous factors, such as local epidemic features, the ability of information or screening campaigns to reach specific subgroups, the availability and cost of screening tests, and the capacity of healthcare providers to identify the first signs of the disease. A significant association between perceived HIV-related stigma and late presentation for HIV/AIDS care in low- and middle-income countries has also been demonstrated [22].

In this context, analyzing the prevalence and causes of delayed presentation for care is definitely of importance.

More than 10,000 HIV^+^ individuals were included in our study. The level of late presentation was high, approximately 45%, and comparable to percentages in other countries [14, 19, 23]. It should be noted that this number is likely underestimated, as CD4 cell count at baseline was rarely performed in the DRC, particularly during recent years [21].

Although the mean percentage of LP was high, we showed an encouraging, strong and persistent diminution of LP over time. The impact of the phasing out of CD4 cell count measurement seems minimal, as the percentage of LP similarly decreased when considering only clinical stages. The reduction of LP over time is thus related to an earlier identification of HIV^+^ individuals. A similar diminution of LP in recent years has been observed in Georgia [24]. Interestingly, the decline in late presentation was thought to be associated with improving HIV testing services in the country, but it was also associated with an increased incidence of HIV infection among men who have sex with men (MSM) diagnosed early, showing that the local evolution of the epidemic can impact LP [24].

We showed that the risk of being a late presenter increases with age. Older individuals have been repeatedly identified as a subgroup at risk for LP [14]. Healthcare providers may indeed consider HIV testing less often in individuals who are not perceived as being at high risk of HIV, such as older individuals. This is particularly harmful for older individuals, as they would benefit the most from fast ART initiation [25].

The comparison between the two types of screening centers is also very informative. The pediatric center proposes systematic HIV screening in children and relatives in various clinical contexts, including tuberculosis, hospitalizations, visits to hospital emergency services, and sexual abuse. Our study shows the benefit of this systematic HIV screening. The risk of being a late presenter did not differ between males and females. Several socioeconomic factors have previously been shown to be associated with LP [19, 26]. However, we were not able to analyze such factors.

In conclusion, our work emphasized that the frequency of LP for care is decreasing in Kinshasa, DRC. Our results could likely be extended to other countries in sub-Saharan Africa where similar screening strategies and information campaigns have been performed. Efforts have to be continued and strengthened to reach lower numbers. In particular, information and screening approaches should target specific subgroups, such as older individuals. Voluntary testing should be encouraged through education campaigns targeting neglected groups with a higher risk of LP. More comprehensive data at diagnosis would be helpful in deciphering factors associated with LP. Data should ideally systematically address the reasons for delayed diagnosis. This will hopefully result in a lower rate of LP and ultimately in better treatment outcomes as well as reduced HIV transmission.

## Data Availability

All data are presented in the manuscript.
